# Anterior knee pain and functional outcome following different surgical techniques for tibial nailing: a systematic review

**DOI:** 10.1007/s00068-020-01458-2

**Published:** 2020-08-09

**Authors:** Mandala S. Leliveld, Michael H. J. Verhofstad, Eduard Van Bodegraven, Jules Van Haaren, Esther M. M. Van Lieshout

**Affiliations:** Trauma Research Unit, Department of Surgery, Erasmus MC, University Medical Center Rotterdam, P.O. Box 2040, 3000 CA Rotterdam, The Netherlands

**Keywords:** Infrapatellar tibial nailing, Suprapatellar tibial nailing, Outcome

## Abstract

**Purpose:**

The aim of this systematic review was to compare knee pain and function after tibial nail insertion through an infrapatellar, semi-extended and suprapatellar technique.

**Methods:**

A search was carried out to identify articles with an exact description of the method used for insertion of the tibial nail and description of the outcome parameters (knee pain or function). Data on study design, population, rate and severity of anterior knee pain and function scores were extracted. Pooled rates and scores were calculated.

**Results:**

67 studies with 3,499 patients were included. The pooled rate of patients with anterior knee pain was 38% (95% CI 32–44) after nail insertion through an infrapatellar approach and 10% (95% CI 1–26) after insertion through a suprapatellar approach. Pooled analysis was not possible for the semi-extended technique. Knee pain scores as measured by visual analogue score (0–10) ranged from 0.2 (95% CI − 0.1–0.5) for general knee pain to 3.7 (95% CI 1.3–6.1) for pain during kneeling. Pooled estimates for the Lysholm score were 87 points (range 77–97) for the infrapatellar technique and 85 points (range 82–85) for the suprapatellar technique. Iowa Knee scores were 94 (range 86–96) and Anterior Knee Pain Scale scores were 76 (range 75–80) after infrapatellar nail insertion.

**Discussion:**

Depending on the technique used, the proportion of patients with knee pain after tibial nailing varied between 10 and 38%. The actual measured knee pain scores were, however, surprisingly low. Knee function was good for both the infra- and suprapatellar technique.

**Electronic supplementary material:**

The online version of this article (10.1007/s00068-020-01458-2) contains supplementary material, which is available to authorized users.

## Introduction

Diaphyseal fractures of the tibia are commonly treated with an intramedullary nail. The infrapatellar approach is most commonly used. However, hyperflexion of the knee during this procedure is associated with an increased risk of valgus and procurvatum deformities in proximal third tibial shaft fractures. In an attempt to address this problem, a semi-extended technique has been developed [1, 2], of which also a subcutaneous variant exists [3]. For the same reasons, the suprapatellar approach has been introduced [4–6]. For this approach, an incision is made just proximal to the superior pole of the patella and the nail is inserted through the patellofemoral joint. The first clinical studies have suggested favorable outcomes associated with a suprapatellar approach [4, 5, 7–9]. The concern of potential damage to the cartilage of the patellofemoral joint remains a significant drawback, although rates of anterior knee pain after this procedure seem lower than seen after the infrapatellar approach [5, 7, 9].

Although all techniques for nail insertion have been proven feasible, a comparison of their rates of anterior knee pain and functional outcome is lacking. The aim of this systematic review and pooled analysis was, therefore, to compare these parameters between different techniques for tibial nail insertion. This information gives perspective to the patient’s rehabilitation after tibial nailing and can aid surgeons in their decision to choose between these surgical techniques.

## Patients and methods

The following databases were searched on December 19, 2018: Embase, Medline (OvidSP), Web of Science, Cochrane Central Register of Controlled Trials (CENTRAL), and Google Scholar. Searched items consisted of terms related to tibia shaft, intramedullary nailing and terms related to pain and function (for full search strategy, see Supplementary data).

Titles and abstracts were screened independently by three reviewers (MSL, JVH, and EAVB). Inconsistencies were resolved by consensus. Studies were included if they met the following inclusion criteria: (1) tibial shaft fracture treated with intramedullary nailing, (2) description of the surgical method used for insertion of the tibial nail (infrapatellar, (subcutaneous) semi-extended or suprapatellar; insertion through patellar tendon, medial or lateral to patellar tendon; use of longitudinal or transverse incision) and (3) primary data for at least one of the outcome parameters (knee pain, function). No limitations on language were considered and only studies from 1990 onwards were included. Studies were excluded if no full-text version was available after contacting corresponding authors. Studies encompassing patients with intra-articular fractures (i.e., tibia plateau or pilon fracture) or only patients with ipsilateral fractures (*i.e*., patients with a floating knee), studies that described only pathological fractures or those with a population aged < 18 years, were excluded. Case reports and letters to or from the editor were also excluded. Reference lists of review articles and eligible studies were examined for additional studies that may have been missed.

Randomized controlled trials (RCTs) and cohort studies were found to be eligible. Patient groups of comparative studies that were treated with the same incision were taken together; the pooled study population was considered one cohort over which knee pain rate, pain and functional scores were calculated.

Two reviewers (MSL and EAVB) independently assessed the methodological quality of the studies using the MINORS (Methodological Index for Non-Randomized Studies) scale [10] (see Supplementary Materials), the global ideal score being 16 for non-comparative studies and 24 for comparative studies.

Data were independently extracted in duplicate by three reviewers (MSL, JVH, and EAVB) using a standardized data sheet. Discrepancies were resolved by consensus. The following data were extracted for each publication: name of first author, publication year, population size and age, percentage of polytrauma patients and patients with ipsilateral fractures, the approach used, the rate of anterior knee pain, the pain scores, functional outcome scores, and the moment at which these measurements were done. When measurements were done at different time points, the scores at 12 months were used for calculation.

Analyses were performed using MedCalc Statistical Software (version 17.6; MedCalc Software bvba, Ostend, Belgium; https://www.medcalc.org; 2017). The rates of anterior knee pain were computed for each study and expressed as percentage. Visual Analog Scales (VAS) with a scale 0–100 were divided by 10 to compare them with 10 cm VAS and 10-point Numeric Rating Scales (NRS). Heterogeneity of the data was assessed using the Cochrane χ^2^ *Q*-test (significance set at *p* < 0.10) and *I*^2^ statistic. Outcomes for cohorts with the same surgical approach were pooled if data were available for at least two groups. A random effects model was used if the *I*^2^ statistic was > 40%; a fixed-effect model was used if it was < 40%. For comparative studies, the relative risk (RR_transpatellar/parapatellar medial_ and RR_infrapatellar/suprapatellar_) was determined for binomial variables and a mean difference for continuous variables. Pooled estimates and relative risks are reported with their 95% confidence interval.

## Results

**Fig. 1:**
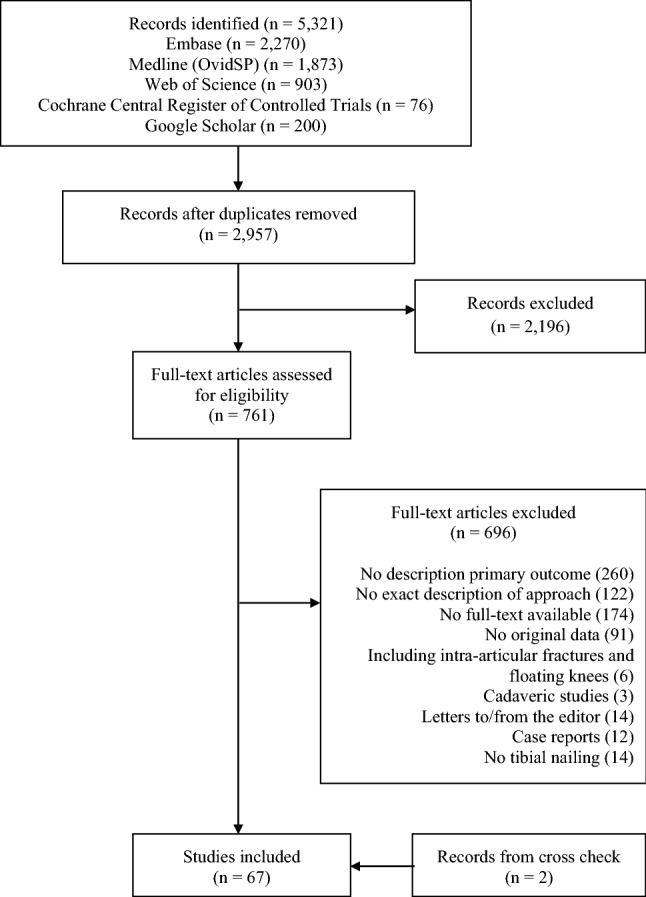
Study flowchart

The literature search identified 6184 potentially eligible studies. After removal of the duplicates (2737 studies) and applying the inclusion and exclusion criteria, 77 studies remained for analysis (Fig. 1).

In the majority of the studies, the infrapatellar approach was described [4, 7–9, 11–78]). Six studies reported on the suprapatellar approach [4, 5, 7–9, 79–83] and one on the semi-extended technique [66]. There were 17 randomized trials [7, 9, 19, 23, 26, 30, 48, 49, 52, 54, 57, 59–61, 64, 68, 71] of which five compared different methods for tibial nailing [7, 9, 26, 49, 71], 14 prospective studies [5, 11, 14, 28, 31, 34, 37, 39, 46, 55, 70, 72, 73, 83] and 45 retrospective studies [4, 8, 12, 13, 15–18, 20–22, 24, 25, 27, 29, 32, 33, 35, 36, 38, 40–45, 47, 50, 51, 53, 56, 58, 62, 63, 66, 67, 69, 74, 76, 78–82, 84]. The mean follow-up ranged from 8 [63] to 94 [28] months. In the majority of the papers, it was clearly stated that the study population did not comprise any polytrauma patients [7–9, 22–26, 28, 32, 33, 36, 43, 44, 48, 51, 53, 55, 60, 63, 64, 66, 68, 71, 74, 79, 81, 84]. However, 23 studies included multiple injured patients in their population [5, 11–14, 16, 18, 19, 27, 30, 34, 35, 37–39, 41, 52, 54, 62, 65, 72, 80, 82], ranging from 4% [38] to 100% [80]. In those articles that included patients with ipsilateral fractures [5, 12, 19, 30, 32, 34, 35, 46, 52, 54, 62, 67, 80, 82], the proportion of ipsilateral fractures was between 3% [19] and 56% [80]. These patients were excluded in 29 studies [7–9, 22–26, 28, 33, 36, 38, 43–45, 48, 51, 53, 55, 60, 64, 66, 68, 71–73, 77, 79, 81]. The moment at which data on anterior knee pain or function were conveyed, was documented in 28 studies [4, 5, 7, 9, 18–20, 25, 26, 28, 30, 42, 45, 49, 51, 53, 54, 56, 58, 60, 66, 70–73, 81, 83] and ranged from 3 months [71] to 94 months [28].

## Anterior knee pain

### Pain rate

The pooled percentage of patients with anterior knee pain after intramedullary nailing was 36% (95% CI 31–42) after use of the infrapatellar approach and 10% (95% CI 2–22) after the suprapatellar approach (Table 1). The relative risk of anterior knee pain after tibial nailing was 1.3 (95% CI 0.9–2.0) when comparing the infrapatellar and suprapatellar techniques [4, 7, 9, 81].

**Table 1:** Pooled pain rates per (sub)group

Parameter	(Sub)group	Studies (*N*)	Population (*N*)	*Q* (*p*-value)	*I*^2^ (95% CI)	Pooled estimate (95% CI)
Pain (%)	Infrapatellar technique	51	2853	612.3 (< 0.0001)	92 (90–93)	38 (32–44)
	Suprapatellar technique	5	174	29.2 (< 0.0001)	86 (70–94)	10 (1–26)

### Pain scores

**Fig. 2:**
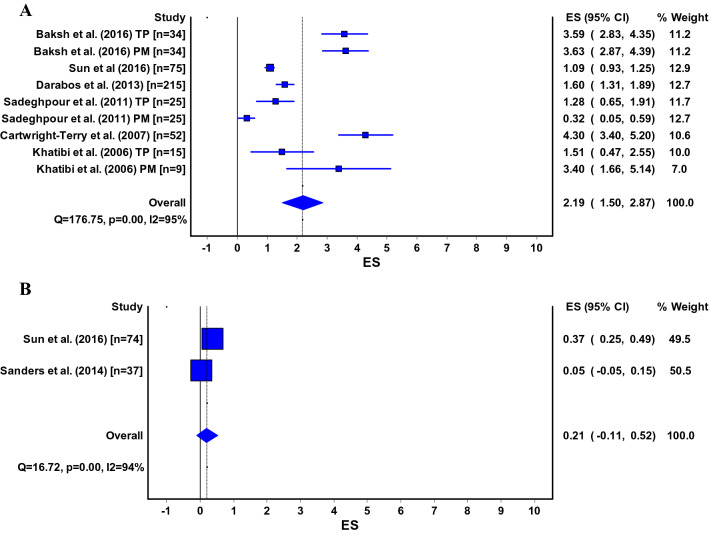
**a**, **b** ES, effect size (pooled estimate for Visual Analogue Score); 95% CI, 95% Confidence Interval; Q, Cochran’s Q-statistic for study heterogeneity; I2, statistic for study heterogeneity; numbers indicate the number of patients in each study or subgroup; TP, transpatellar approach; PM, parapatellar medial approach

Six different scales were used for measuring the severity of anterior knee pain (Table 2). For the majority of the studies, it was not documented on how data on knee pain were retrieved. Pooled estimates for knee pain (VAS 0–10) were 2.5 (95% CI 1.5–3.4) for the infrapatellar technique and 0.4 (95% CI 0.0–0.7) for the suprapatellar technique (Fig. 2a, b). Pain scores for specific (daily) activities could only be pooled for the infrapatellar technique. Kneeling was reported as most painful (VAS 3.7; 95% CI 1.3–6.1) [26, 53, 58]. Pain scores for other activities were described in two studies [26, 53]: 0.3 (95% CI − 0.1–0.7) in rest, 0.6 (95% CI − 0.0–1.1) for prolonged sitting with knees bend, 0.5 (95% CI 0.01–1.0) during walking, 1.0 (95% CI 0.0–2.1) for running, 1.6 (95% CI 0.5–2.7) while squatting, 1.1 (95% CI 0.2–2.1) for ascending stairs and 0.9 (95% CI − 0.1–1.9) for descending stairs.

**Table 2:** Different instruments used for measuring knee pain

Instrument used to measure knee pain	*N* studies
Unspecified [11–16, 19–23, 25, 27, 29, 30, 32, 34–37, 40, 41, 44, 47, 50, 52, 59, 62, 64, 65, 69, 71]	32
VAS 0–10 [5, 7, 9, 24, 38, 46, 49, 55, 56, 63, 70]	11
VAS 0–100 [26, 33, 53, 58, 60]	5
Direct questioning [20, 28, 45, 51, 54]	5
NRS 0–10 [17, 42, 67]	3
Oxford Knee Score (pain component) [8]	1
Lysholm Knee Score (pain component) [72]	1
Kujala or Anterior Knee Pain Scale (pain component) [4]	1

## Function

### General function lower extremity

To measure the lower extremity function in general, the Tegner Activity Score [14, 23, 26, 53, 77], Lower Extremity Functional Score [35], and Musculoskeletal Function Assessment [54] were used. The pooled analysis for the Tegner Activity score was 3.9 (95% C.I. 3.6–4.2) for the infrapatellar technique [53].

### Knee function

**Fig. 3:**
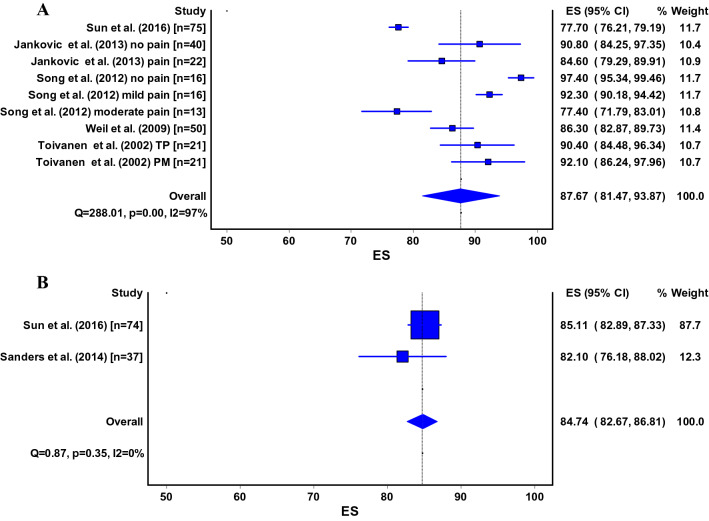
**a**, **b** ES, effect size (pooled estimate for Lysholm score); 95% CI, 95% Confidence Interval; Q, Cochran’s Q-statistic for study heterogeneity; I2, statistic for study heterogeneity; numbers indicate the number of patients in each study or subgroup; TP, transpatellar approach; PM, parapatellar medial approach Pooled estimates for the other surgical methods could not be calculated and are thus not shown

**Fig. 4:**
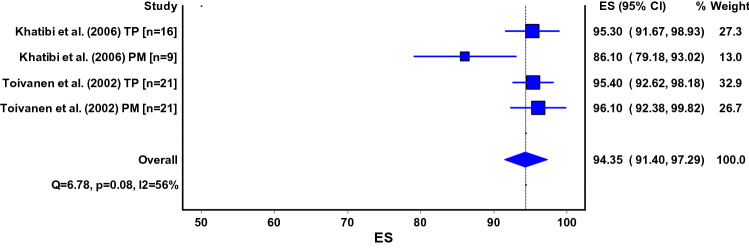
ES, effect size (pooled estimate for Iowa knee score); 95% CI, 95% Confidence Interval; Q, Cochran’s Q-statistic for study heterogeneity; I2, statistic for study heterogeneity; numbers indicate the number of patients in each study or subgroup; TP, transpatellar approach; PM, parapatellar medial approach Pooled estimates for the other surgical methods could not be calculated and are thus not shown

**Fig. 5:**
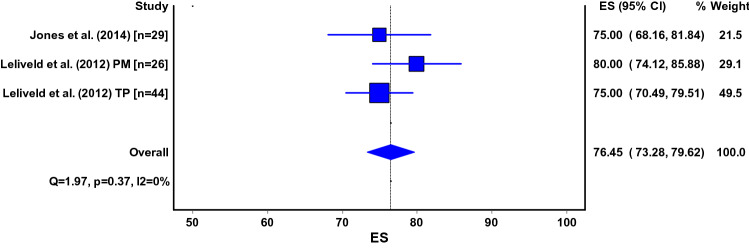
ES, effect size (pooled estimate for Anterior Knee Pain Scale); 95% CI, 95% Confidence Interval; Q, Cochran’s Q-statistic for study heterogeneity; I2, statistic for study heterogeneity; numbers indicate the number of patients in each study or subgroup; TP, transpatellar approach; PM, parapatellar medial approach. Pooled estimates for semi-extended technique could not be calculated and are thus not shown

The Lysholm Scale [5, 9, 26, 45, 53, 56, 66, 73, 75, 77, 79–81, 83, 84], Iowa Knee Score [24, 26, 33, 48], (Kujala) Anterior Knee Pain Scale (AKPS) [4, 51, 80, 81], Functional Anterior Knee Pain Score [38, 75, 80], Oxford Knee Score [8, 80] and International Knee Documentation Committee (IKDC Questionnaire) [80] were used for measuring the knee function after tibial nailing. Pooled estimates for the Lysholm Scale were 87 points (95% CI 81–94) for the infrapatellar technique and 85 points (95% CI 83–87) for the suprapatellar technique (Fig. 3a, b). Pooled analysis for the Iowa Knee Score (Fig. 4) was only possible for the infrapatellar technique and was 94 points (95% CI 91–97) (Fig. 4). Pooled estimates for the Anterior Knee Pain Scale (or Kujala) were 79 points (95% CI 76–83) for the infrapatellar technique and 79 points (95% CI 71–86) for the suprapatellar technique (Fig. 5a, b).

### Ankle function

To measure ankle function, the following instruments were used: AOFAS ankle–hindfoot scoring system [67, 77, 78, 82], Iowa Ankle Score (also known as Merchant and Dietz Ankle Function Score) [24, 31, 33, 36, 48], Olerud and Molander Ankle Score [36, 76, 82], Mazur Ankle Score [59], and Foot Function Index [54]. Pooled estimates for the AOFAS ankle–hindfoot scoring system and Iowa Ankle Score were 91 (95% CI 87–93) and 92 (95% CI 89–96) for the infrapatellar and suprapatellar technique, respectively.

## Quality of life

**Fig. 6:**
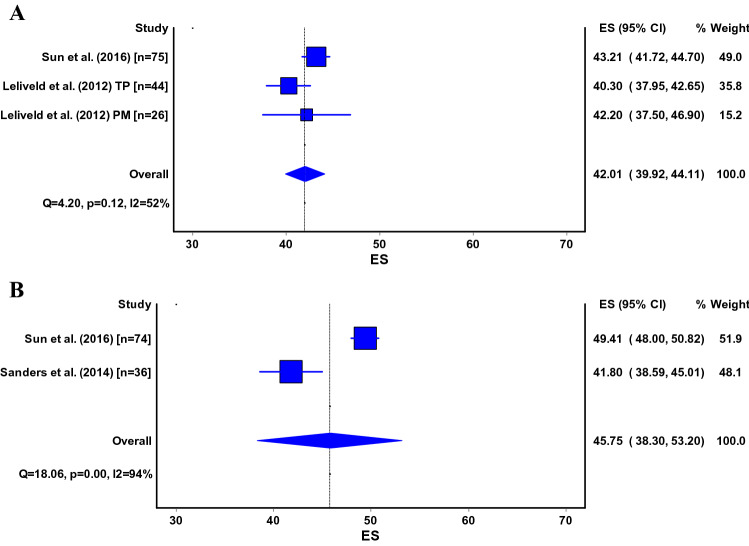
**a**, **b** SF-36, Short Form-36; PCS, physical component score; ES, effect size (pooled estimate for PCS); 95% CI, 95% Confidence Interval; Q, Cochran’s Q-statistic for study heterogeneity; I2, statistic for study heterogeneity; numbers indicate the number of patients in each study or subgroup; TP, transpatellar approach; PM, parapatellar medial approach. Pooled estimates for the semi-extended technique could not be calculated and are thus not shown

**Fig. 7:**
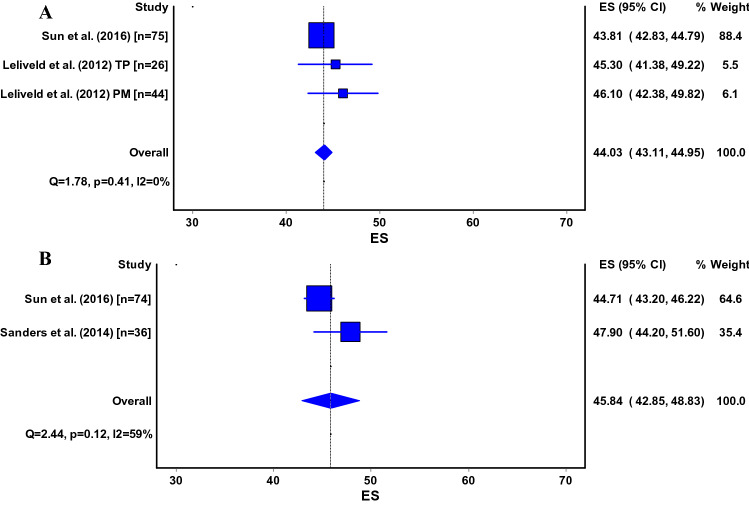
**a**, **b** SF-36, Short Form-36; MCS, mental component score; ES, effect size (pooled estimate for MCS); 95% CI, 95% Confidence Interval; Q, Cochran’s Q-statistic for study heterogeneity; I2, statistic for study heterogeneity; numbers indicate the number of patients in each study or subgroup; TP, transpatellar approach; PM, parapatellar medial approach. Pooled estimates for the semi-extended technique could not be calculated and are thus not shown

The Short Form-36 (SF-36) [5, 7, 9, 24, 43, 48, 51, 61, 67, 80, 83], SF-12 [4], EQ5D [60], and the Nottingham Health Profile [23, 60] were used to measure quality of life after tibial nailing. The pooled estimates could only be calculated for the physical and mental component score (PCS and MCS) of the SF-36. The PCS was 42 (95% CI 40–44) for the infrapatellar technique and 46 (95% CI 41–51) for the suprapatellar technique (Fig. 6a, b). The pooled estimate for the MCS was 44 (95% CI 43–45) for the infrapatellar technique and 48 (95% CI 44–52) for the suprapatellar technique (Fig. 7a, b).

## Discussion

The aim of this systematic review was to compare knee pain and function after tibial nail insertion through different surgical methods. For the infrapatellar approach, the proportion of patients with anterior knee pain in the current review was 36%. The percentage found for the suprapatellar technique was 10%. The documented general knee pain scores (VAS/NRS 0–10) were, however, surprisingly low for both techniques (2.5 for the infrapatellar technique and 0.4 for the suprapatellar technique). For the infrapatellar technique, pain scores during common daily activities were also low, except for kneeling (range 3.2–4.7). Knee function was good for both the infra- and suprapatellar techniques.

The pooled proportion of 36% of patients with knee pain is lower than the much quoted percentage of 47.4% from the systematic review by Katsoulis et al. [85], but it is still a substantial percentage. Although many patients report pain, pooled estimates were high for the Lysholm score, Iowa Knee score and AKPS. The scope of most knee function scores is limited to patients with osteoarthritis or those receiving total knee replacements. For fractures around the knee, there is currently no validated, reliable, and reproducible outcome measure. For patients with tibia fractures only, the disease-specific Short Musculoskeletal Function Assessment (SMFA) and the generic measure SF-36 have been demonstrated responsive and valid [86]. Both assess the general functional status of patients and how bothered they are by functional problems without focus on knee function and knee pain. Since outcome scoring is vital in the accurate evaluation and comparison of interventions, what knee scoring system should we use to measure knee pain and/or function after tibial nailing? The Lysholm Score and Iowa Knee Score [24, 26, 33, 48] are the most commonly used for this cause, but neither is validated for this specific patient population. Validation of (at least one of) these questionnaires in a patient population that include tibial fractures is, therefore, needed.

One limitation of this systematic review is the lack of randomized controlled trials (RCT) comparing different methods in tibial nailing. Only two RCTs compared nail insertion through the patellar tendon with insertion medial to the patellar tendon [26, 49] and two other RCTs compared an infrapatellar and suprapatellar technique [7, 9]. Furthermore, most studies lack information on how pain as an outcome parameter was acquired, as did information at the point of time at which the parameter was measured. The proportion of patients with knee pain might well be higher within the first months after surgery than years later. This should be taken into account when interpreting such percentages. Pain scores and functional outcome measurements can additionally be affected by the presence of other injuries. Therefore, outcome measures from studies that included multiple injured patients or patients with ipsilateral fractures must also be interpreted with caution.

Overall, adequate reporting of outcome measures was poor. Besides, the previously mentioned lack of how and when measurements were taken, the standard deviation for mean pain or functional scores was not always provided. Furthermore, some authors chose to report scores only in terms of excellent, good, fair etc., without mentioning an overall score. The quality of a systematic review, such as the current review, depends on the quality of the underlying studies and although it is the authors’ responsibility to report their data adequate and complete, it would be helpful if journal reviewers and editors would ask for any missing information.

## Conclusion

The question whether one surgical approach for tibial nailing is superior to another cannot be answered due to limited availability of adequate data. One can conclude though that in terms of anterior knee pain, the suprapatellar technique has the lowest proportion (10.0%) of patients with this complaint. Overall, general knee pain scores are low (range 0.2–2.7). Knee function was good for both the infra- and suprapatellar techniques.

## Electronic supplementary material

Below is the link to the electronic supplementary material.

Supplementary file (PDF 262 kb)

## Data Availability

Not applicable.

## References

[1] Tornetta P, Collins E (1996). Semiextended position for intramedullary nailing of the proximal tibia. Clin Orthop Relat Res.

[2] Kubiak EN, Widmer BJ, Horwitz DS (2010). Extra-articular technique for semiextended tibial nailing. J Orthop Trauma.

[3] Morandi M, Banka T, Gaiarsa GP, Guthrie ST, Khalil J, Hoegler J (2010). Intramedullary nailing of tibial fractures: review of surgical techniques and description of a percutaneous lateral suprapatellar approach. Orthopedics..

[4] Jones M, Parry M, Whitehouse M, Mitchell S (2014). Radiologic outcome and patient-reported function after intramedullary nailing: A comparison of the retropatellar and infrapatellar approach. J Orthop Trauma.

[5] Sanders RW, DiPasquale TG, Jordan CJ, Arrington JA, Sagi HC (2014). Semiextended intramedullary nailing of the tibia using a suprapatellar approach: radiographic results and clinical outcomes at a minimum of 12 months follow-up. J Orthop Trauma.

[6] Jakma T, Reynders-Frederix P. Insertion of intramedullary nails from the suprapatellar pouch for proximal tibial shaft fractures. A technical note. Acta Orthop Belg. actaorthopaedica.be; 2011. 22308632

[7] Chan DS, Serrano R, Griffing R, Steverson B, Infante A, Watson D (2015). Supra- versus Infra-patellar tibial nail insertion: a prospective, randomized control pilot study. J Orthop Trauma.

[8] Courtney PM, Boniello A, Donegan D, Ahn J, Mehta S (2015). Functional knee outcomes in infrapatellar and suprapatellar tibial nailing: does approach matter?. Am J Orthop.

[9] Sun Q, Nie XY, Gong JP, Wu JZ, Li RL, Ge W (2016). The outcome comparison of the suprapatellar approach and infrapatellar approach for tibia intramedullary nailing. Int Orthop.

[10] Slim K, Nini E, Forestier D, Kwiatkowski F, Panis Y, Chipponi J (2003). Methodological index for non-randomized studies (minors): development and validation of a new instrument. ANZ J Surg.

[11] Court-Brown CM, Christie J, McQueen MM. Closed intramedullary tibial nailing. Its use in closed and type I open fractures. J Bone Jt Surg Ser B. 1990;72(4):605–11. 10.1302/0301-620X.72B4.2380211 2380211

[12] O'Beirne J, Seigne P, McElwain JP (1992). Interlocking intramedullary nailing for the treatment of tibial fractures. Ir J Med Sci.

[13] Anglen JO, Blue JM (1995). A comparison of reamed and unreamed nailing of the tibia. J Trauma Inj Infect Crit Care.

[14] Krettek C, Schandelmaier P, Tscherne H (1995). Nonreamed interlocking nailing of closed tibial fractures with severe soft tissue injury. Clin Orthop Relat Res.

[15] Orfaly R, Keating JF, O'Brien PJ (1995). Knee pain after tibial nailing: Does the entry point matter?. J Bone Jt Surg Ser B.

[16] Haddad FS, Desai K, Sarkar JS, Dorrell JH (1996). The AO unreamed nail: Friend or foe. Injury.

[17] Court-Brown CM, Gustilo T, Shaw AD (1997). Knee pain after intramedullary tibial nailing: its incidence, etiology, and outcome. J Orthop Trauma.

[18] Keating JF, O'Brien PI, Blachut PA, Meek RN, Broekhuyse HM (1997). Reamed interlocking intramedullary nailing of open fractures of the tibia. Clin Orthop Relat Res.

[19] Keating JF, Obrien PJ, Blachut PA, Meek RN, Broekhuyse HM (1997). Locking intramedullary nailing with and without reaming for open fractures of the tibial shaft - a prospective, randomized study. J Bone Joint Surg-Am.

[20] Keating JF, Orfaly R, O'Brien PJ (1997). Knee pain after tibial nailing. J Orthop Trauma.

[21] Lovell ME, Sharma S, Allcock S, Hardy SK (1998). Insertion site for intramedullary tibial nails, and its relationship to anterior knee pain. Knee.

[22] Martínez Martin AA, Panisello Sebastián JJ, Herrera Rodríguez A, Domingo Cebollada J, Calvo DA (1998). Treatment of closed fractures of the tibial diaphysis by unreamed intramedullary nailing. Rev Ortop Traumatol.

[23] Karladani AH, Granhed H, Edshage B, Jerre R, Styf J (2000). Displaced tibial shaft fractures: a prospective randomized study of closed intramedullary nailing versus cast treatment in 53 patients. Acta Orthop Scand.

[24] Dogra AS, Ruiz AL, Marsh DR (2002). Late outcome of isolated tibial fractures treated by intramedullary nailing: The correlation between disease-specific and generic outcome measures. J Orthop Trauma.

[25] Shannon FJ, Mullett H, O'Rourke K (2002). Unreamed intramedullary nail versus external fixation in grade III open tibial fractures. J Trauma.

[26] Toivanen JAK, Vaisto O, Kannus P, Latvala K, Honkonen SE, Jarvinen MJ (2002). Anterior knee pain after intramedullary nailing of fractures of the tibial shaft - A prospective, randomized study comparing two different nail-insertion techniques. J Bone Joint Surg-Am.

[27] Bombaci H, Güneri B, Görgeç M, Kafadar A (2004). A comparison between locked intramedullary nailing and plate-screw fixation in the treatment of tibial diaphysis fractures. Acta Orthop Traumatol Turc.

[28] Fankhauser F, Seibert FJ, Boldin C, Schatz B, Lamm B (2004). The unreamed intramedullary tibial nail in tibial shaft fractures of soccer players: a prospective study. Knee Surg Sports Traumatol Arthrosc.

[29] Al Hussainy HAJ, Deeb A, Choudhary AK (2005). Anterior knee pain following intramedullary nailing of tibial shaft fractures: Does bony portal point in the sagittal plane affect the outcome?. Eur J Orthop Surg Traumatol.

[30] Bråten M, Helland P, Groøntvedt T, Aamodt A, Benum P, Mølster A (2005). External fixation versus locked intramedullary nailing in tibial shaft fractures: A prospective, randomised study of 78 patients. Arch Orthop Trauma Surg.

[31] Fan CY, Chiang CC, Chuang TY, Chiu FY, Chen TH (2005). Interlocking nails for displaced metaphyseal fractures of the distal tibia. Injury.

[32] Djahangiri A, Garofalo R, Chevalley F, Leyvraz PF, Wettstein M, Borens O (2006). Closed and open grade I and II tibial shaft fractures treated by reamed intramedullary nailing. Med Princ Pract.

[33] Khatibi H, Jah AAE, Zaeem MMS, Moghaddam AK (2006). Knee and ankle function after displaced non-isolated fractures of the tibial shaft, a retrospective comparison between plate fixation and intramedullary nailing. Eur J Orthop Surg Traumatol.

[34] Lin J (2006). Effectiveness of completely round nails with both-ends-threaded locking screws for tibial shaft fractures. J Trauma Inj Infect Crit Care.

[35] Vidyadhara S, Sharath KR (2006). Prospective study of the clinico-radiological outcome of interlocked nailing in proximal third tibial shaft fractures. Injury.

[36] Yang SW, Tzeng HM, Chou YJ, Teng HP, Liu HH, Wong CY (2006). Treatment of distal tibial metaphyseal fractures: Plating versus shortened intramedullary nailing. Injury.

[37] Babis GC, Benetos IS, Karachalios T, Soucacos PN (2007). Eight years' clinical experience with the Orthofix® tibial nailing system in the treatment of tibial shaft fractures. Injury.

[38] Cartwright-Terry M, Snow M, Nalwad H (2007). The severity and prediction of anterior knee pain post tibial nail insertion. J Orthop Trauma.

[39] Lee YS, Lo TY, Huang HL (2008). Intramedullary fixation of tibial shaft fractures: A comparison of the unlocked and interlocked nail. Int Orthop.

[40] Mohammed A, Saravanan R, Zammit J, King R (2008). Intramedullary tibial nailing in distal third tibial fractures: Distal locking screws and fracture non-union. Int Orthop.

[41] Burç H, Dursun M, Orhun H, Gürkan V, Bayhan I (2009). Treatment of adult tibial diaphysis fractures with reamed and locked intramedullary nailing. Acta Orthop Traumatol Turc.

[42] Kim KTSSK, Kang MS, Jin X, Lee CW, Wang L. Anterior knee pain after intramedullary tibial nailing. J Korean Orthop Assoc. 2009;44:61–7.

[43] Nascimento OR, Cemin FS, de Morais M, Barroco RD, Fujiki EN, Milani C (2009). Assessment of quality of life in patients with tibial fractures. Acta Ortop Bras.

[44] Uzümcügil O, Doǧan A, Yalçinkaya M, Kabukçuoǧlu YS (2009). The relationship between anterior knee pain occurring after tibial intramedullary nailing and the localization of the nail in the proximal tibia. Acta Orthop Traumatol Turc.

[45] Weil YA, Gardner MJ, Boraiah S, Helfet DL, Lorich DG (2009). Anterior knee pain following the lateral parapatellar approach for tibial nailing. Arch Orthop Trauma Surg.

[46] Darabos N, Bajs ID, Rutic Z, Darabos A, Poljak D, Dobsa J (2011). Nail position has an influence on anterior knee pain after tibial intramedullary nailing. Coll Antropol.

[47] Demirtaş A, Azboy I, Durakbaşa MO, Uçar BY, Mercan AS, Çakir IA (2011). The relationship between the quadriceps muscle strength and the anterior knee pain occurring after locked intramedullary nailing for tibial diaphysis fractures. Eklem Hast Cerrahisi.

[48] Maity A, Mondal A, Mondal BC, Roy DS (2011). Prospective comparative study of functional outcome of treatment of tibial shaft fracture in adult by cast versus intramedullary nailing. J Clin Orthop Traum.

[49] Sadeghpour A, Mansour R, Aghdam HA, Goldust M (2011). Comparison of trans patellar approach and medial parapatellar tendon approach in tibial intramedullary nailing for treatment of tibial fractures. J Pak Med Assoc.

[50] Hernandez-Vaquero D, Suarez-Vazquez A, Iglesias-Fernandez S, Garcia-Garcia J, Cervero-Suarez J (2012). Dynamisation and early weight-bearing in tibial reamed intramedullary nailing: its safety and effect on fracture union. Injury.

[51] Leliveld MS, Verhofstad MHJ (2012). Injury to the infrapatellar branch of the saphenous nerve, a possible cause for anterior knee pain after tibial nailing?. Injury.

[52] Malik NI, Iqbal P, Rafi I, Shah WA (2012). Intramedullary nailing for open fractures of the tibial shaft. Med Forum Monthly.

[53] Song SY, Chang HG, Byun JC, Kim TY (2012). Anterior knee pain after tibial intramedullary nailing using a medial paratendinous approach. J Orthop Trauma.

[54] Vallier HA, Cureton BA, Patterson BM (2012). Factors influencing functional outcomes after distal tibia shaft fractures. J Orthop Trauma.

[55] Daraboš N, Banić T, Lubina Z, Daraboš A, Bilić V, Sabalić S (2013). Precise nail tip positioning after tibial intramedullary nailing prevents anterior knee pain. Int Orthop.

[56] Jankovic A, Korac Z, Bozic NB, Stedul I. Influence of knee flexion and atraumatic mobilisation of infrapatellar fat pad on incidence and severity of anterior knee pain after tibial nailing. Injury. 2013;44(SUPPL.3):S33-S9. doi: 10.1016/s0020-1383(13)70195-5. 10.1016/S0020-1383(13)70195-5 24060016

[57] Prasad M, Yadav S, Sud A, Arora NC, Kumar N, Singh S (2013). Assessment of the role of fibular fixation in distal-third tibia-fibula fractures and its significance in decreasing malrotation and malalignment. Injury.

[58] Chen CY, Lin KC, Yang SW, Tarng YW, Hsu CJ, Renn JH (2014). Influence of nail prominence and insertion point on anterior knee pain after tibial intramedullary nailing. Orthopedics.

[59] Li Y, Jiang X, Guo Q, Zhu L, Ye T, Chen A (2014). Treatment of distal tibial shaft fractures by three different surgical methods: A randomized, prospective study. Int Orthop.

[60] Ramos T, Eriksson BI, Karlsson J, Nistor L (2014). Ilizarov external fixation or locked intramedullary nailing in diaphyseal tibial fractures: A randomized, prospective study of 58 consecutive patients. Arch Orthop Trauma Surg.

[61] Rodrigues FL, de Abreu LC, Valenti VE, Valente AL, da Costa Pereira Cestari R, Pohl PHI, et al. Bone tissue repair in patients with open diaphyseal tibial fracture treated with biplanar external fixation or reamed locked intramedullary nailing. Injury. 2014;45 Suppl 5:S32–5. 10.1016/S0020-1383(14)70018-X 25528622

[62] Kruppa CG, Hoffmann MF, Sietsema DL, Mulder MB, Jones CB (2015). Outcomes after intramedullary nailing of distal tibial fractures. J Orthop Trauma.

[63] Kumar KH (2015). A Study of the Management of Open Fractures of Tibia by Unreamed Interlocking Nail. J Evol Med Dent Sci-JEMDS.

[64] Meena RC, Meena UK, Gupta GL, Gahlot N, Gaba S (2015). Intramedullary nailing versus proximal plating in the management of closed extra-articular proximal tibial fracture: a randomized controlled trial. J Orthop Traumatol.

[65] Zhu DC, Liu L, Gao F, Li Q, Zhang B (2015). Comparison of closed reduction and expert tibial nailing with open reduction and plate and screw fixation in the treatment of two segmental tibial fractures. Chin J Traumatol Eng Ed.

[66] Bakhsh WR, Cherney SM, McAndrew CM, Ricci WM, Gardner MJ. Surgical approaches to intramedullary nailing of the tibia: Comparative analysis of knee pain and functional outcomes. Injury. 2016. 10.1016/j.injury.2015.12.025 26830120

[67] Barcak E, Collinge CA (2016). Metaphyseal distal tibia fractures: a cohort, single-surgeon study comparing outcomes of patients treated with minimally invasive plating versus intramedullary nailing. J Orthop Trauma.

[68] Fang JH, Wu YS, Guo XS, Sun LJ (2016). Comparison of 3 minimally invasive methods for distal tibia fractures. Orthopedics.

[69] Soraganvi PC, Anand-Kumar BS, Rajagopalakrishnan R, Praveen-Kumar BA (2016). Anterior knee pain after Tibial intra-medullary nailing: is it predictable?. Malays Orthop J.

[70] Ahmad M, Bashir T, Aslam M (2016). Comparison of anterior knee pain following tibia ILN in Patellar tendon split and retraction insertion. Pak J Med Health Sci.

[71] Ahmad S, Ahmed A, Khan L, Javed S, Ahmed N, Aziz A (2016). Comparative analysis of anterior knee pain in transpatellar and medial parapatellar tendon approaches in tibial interlocking nailing. J Ayub Med Coll Abbottabad.

[72] Ranganath KV, Arun HS, Hariprasad S (2016). Surgical management of isolated tibial shaft fractures with closed intramedullary interlocking nail. Int J Sci Study.

[73] Esan O, Ojoawo AO, Ikem IC (2017). Quadriceps strength and anterior knee pain following tibia intramedullary nailing: any clinical relationship?. Niger J Clin Pract.

[74] Duygun F, Aldemir C (2018). Effect of intramedullary nail compression amount on the union process of tibial shaft fracture and the evaluation of this effect with a different parameter. Eklem Hastalik Cerrahisi.

[75] Ozcan C, Turkmen I, Sokucu S (2018). Comparison of three different approaches for anterior knee pain after tibia intramedullary nailing. Eur J Trauma Emerg Surg.

[76] Bisaccia M, Cappiello A, Meccariello L, Rinonapoli G, Falzarano G, Medici A (2018). Nail or plate in the management of distal extra-articular tibial fracture, what is better? Valutation of outcomes. SICOT J.

[77] Camurcu Y, Sofu H, Issin A, Kockara N, Genc E, Cetinkaya M (2017). Is talon tibial intramedullary nailing clinically superior compared to conventional locked nailing?. Eklem Hastalik Cerrahisi.

[78] Yavuz U, Sokucu S, Demir B, Yildirim T, Ozcan C, Kabukcuoglu YS (2014). Comparison of intramedullary nail and plate fixation in distal tibia diaphyseal fractures close to the mortise. Ulus Travma Acil Cerrahi Derg.

[79] Zhang J, Ma JW (2017). Magnetic navigation META-NAIL interlocking intramedullary nailing for tibial shaft fractures via the supra-patellar approach. Chin J Tissue Eng Res.

[80] Cazzato G, Saccomanno MF, Noia G, Masci G, Peruzzi M, Marinangeli M (2018). Intramedullary nailing of tibial shaft fractures in the semi-extended position using a suprapatellar approach: a retrospective case series. Injury.

[81] Ozcan C, Turkmen I, Sokucu S. Comparison of three different approaches for anterior knee pain after tibia intramedullary nailing. 2018. 10.1007/s00068-018-0988-6 30039307

[82] Fu B (2016). Locked META intramedullary nailing fixation for tibial fractures via a suprapatellar approach. Indian J Orthop.

[83] Serbest S, Tiftikci U, Coban M, Cirpar M, Daglar B (2019). Knee pain and functional scores after intramedullary nailing of tibial shaft fractures using a suprapatellar approach. J Orthop Trauma.

[84] Çamurcu Y, Sofu H, Issın A, Koçkara N, Genç E, Çetinkaya M (2017). Is talon tibial intramedullary nailing clinically superior compared to conventional locked nailing?. Eklem Hastalik Cerrahisi.

[85] Katsoulis E, Court-Brown C, Giannoudis PV (2006). Incidence and aetiology of anterior knee pain after intramedullary nailing of the femur and tibia review. J Bone Joint Surg Br.

[86] Busse JW, Bhandari M, Guyatt GH, Heels-Ansdell D, Mandel S, Sanders D (2009). Use of both short musculoskeletal function assessment questionnaire and Short Form-36 among tibial-fracture patients was redundant. J Clin Epidemiol.

