# Supplementary material for: Anterior knee pain and functional outcome following different surgical techniques for tibial nailing: a systematic review
Source: Eur J Trauma Emerg Surg. 2020 Aug 9;47(3):763–72. doi: 10.1007/s00068-020-01458-2 (PMC8187175; doi:10.1007/s00068-020-01458-2)
Supplement: Supplementary file 1 — Supplementary file (PDF 262 kb) [file 68_2020_1458_MOESM1_ESM.pdf]

Embase.com (Embase plus Medline): 2270
('tibia shaft fracture'/de OR 'tibia fracture'/de OR 'distal tibia fracture'/de OR 'proximal tibia fracture'/de OR (tibia* AND fractur*):ab,ti) **AND** ('Bone nail'/de OR 'intramedullary nail'/exp OR 'intramedullary nailing'/de OR (((intramedullar* OR IM OR medullar* OR tibial OR ender) NEAR/4 (nail* OR rod* OR pin* OR fixation* OR osteosynth*)) OR (bone NEAR/3 (nail* OR pin* OR rod*)) OR Micronail* OR Olecranail* OR Targon* OR Fixion* OR Trigen):ab,ti) **AND** ('treatment outcome'/exp OR 'pain'/exp OR 'musculoskeletal function'/exp OR (outcome* OR pain* OR function*):ab,ti)

Medline Epub (OvidSP): 1873
("Tibial Fractures"/ OR (tibia* AND fractur*).ab,ti.) **AND** ("Bone Nails"/ OR "Fracture Fixation, Intramedullary"/ OR (((intramedullar* OR IM OR medullar* OR tibia* OR ender) ADJ4 (nail* OR rod* OR pin* OR fixation* OR osteosynth*)) OR (bone ADJ3 (nail* OR pin* OR rod*)) OR Micronail* OR Olecranail* OR Targon* OR Fixion* OR Trigen).ab,ti.) **AND** (exp "treatment outcome"/ OR exp "pain"/ OR "recovery of function"/ OR (outcome* OR pain* OR function*).ab,ti.)

Cochrane Central (trials): 76
((tibia* AND fractur*):ab,ti) **AND** ((((intramedullar* OR IM OR medullar* OR tibial OR ender) NEAR/4 (nail* OR rod* OR pin* OR fixation* OR osteosynth*)) OR (bone NEAR/3 (nail* OR pin* OR rod*)) OR Micronail* OR Olecranail* OR Targon* OR Fixion* OR Trigen):ab,ti) **AND** ((outcome* OR pain* OR function*):ab,ti)

Web of Science: 903
TS=((tibia* AND fractur*) **AND** (((intramedullar* OR IM OR medullar* OR tibial OR ender) NEAR/3 (nail* OR rod* OR pin* OR fixation* OR osteosynth*)) OR (bone NEAR/2 (nail* OR pin* OR rod*)) OR Micronail* OR Olecranail* OR Targon* OR Fixion* OR Trigen) **AND** (outcome* OR pain* OR function*)**)**

Google Scholar: 200 (sorted on relevance)
"tibia fracture" "intramedullary|IM|medullary|tibial|ender nail|rod|pin|fixation"|"bone nail|pin|rod"|Micronail|Olecranail|Targon|Fixion|Trigen outcome|pain|functioning
